# Neuromodulation: present and emerging methods

**DOI:** 10.3389/fneng.2014.00027

**Published:** 2014-07-15

**Authors:** Song Luan, Ian Williams, Konstantin Nikolic, Timothy G. Constandinou

**Affiliations:** ^1^Department of Electrical and Electronic Engineering, Imperial College LondonLondon, UK; ^2^Center for Bio-Inspired Technology, Institute of Biomedical Engineering, Imperial College LondonLondon, UK

**Keywords:** neuromodulation, neural modulation, neurostimulation, neural stimulation, neuroprosthetics, neural prosthesis

## Abstract

Neuromodulation has wide ranging potential applications in replacing impaired neural function (prosthetics), as a novel form of medical treatment (therapy), and as a tool for investigating neurons and neural function (research). Voltage and current controlled electrical neural stimulation (ENS) are methods that have already been widely applied in both neuroscience and clinical practice for neuroprosthetics. However, there are numerous alternative methods of stimulating or inhibiting neurons. This paper reviews the state-of-the-art in ENS as well as alternative neuromodulation techniques—presenting the operational concepts, technical implementation and limitations—in order to inform system design choices.

## 1. Introduction

Neuromodulation is already a multi-billion dollar industry and is expected to double in the near future (Gofeld, 2014). Alone has seen the launch of the European Human Brain Project and the US Brain Research through Advancing Innovative Neurotechnologies (BRAIN) Initiative—massive collaborative projects which look set to enhance our understanding and create myriad new opportunities in this space.

Today there are 3 main applications for neuromodulation. (1) Prosthetics—devices that replace or improve impaired sensory, motor or cognitive neural function—examples already exist in clinical use such as cochlear implants, and many more are in active development such as retinal and proprioceptive implants (Theogarajan, 2012; Williams and Constandinou, 2013). (2) Therapy—devices to neurally regulate the body's organs for medical benefit—possibly enabling control of insulin release for diabetics or renal salt absorption for people with hypertension (Stanslaski et al., 2012; Famm et al., 2013). It is also used for treatment of epilepsy, depression, traumatic brain injury, Parkinsons disease and obesity (Testerman et al., 2006). (3) Neuroscience research—investigating the function of neurons and neural networks in the peripheral and central nervous system (PNS and CNS)—enhancing or creating new applications for neuromodulation (Fenno et al., 2011).

All of these applications employ neuromodulation—stimulating or blocking the flow of Action Potentials (APs) through the nervous system. Electrical Neural Stimulation (ENS) has historically been the main technique for neuromodulation and has played a crucial role in neuroscience ever since Galvani first demonstrated that neurons could be electrically stimulated in the 18th century. However, recent developments in alternative modulation methods potentially offer significant advantages over ENS and could catalyze a wide expansion of neuromodulation for clinical applications.

This paper presents the current state of the art in neuromodulation methods—describing advantages, limitations, implementations and applicability. It is laid out as follows: section 2 discusses the physics of action potential creation; section 3 describes and contrasts the various methods of achieving neuromodulation; and section 4 summarizes the current state of the art.

## 2. Biophysics of action potential generation

**Figure 1:**
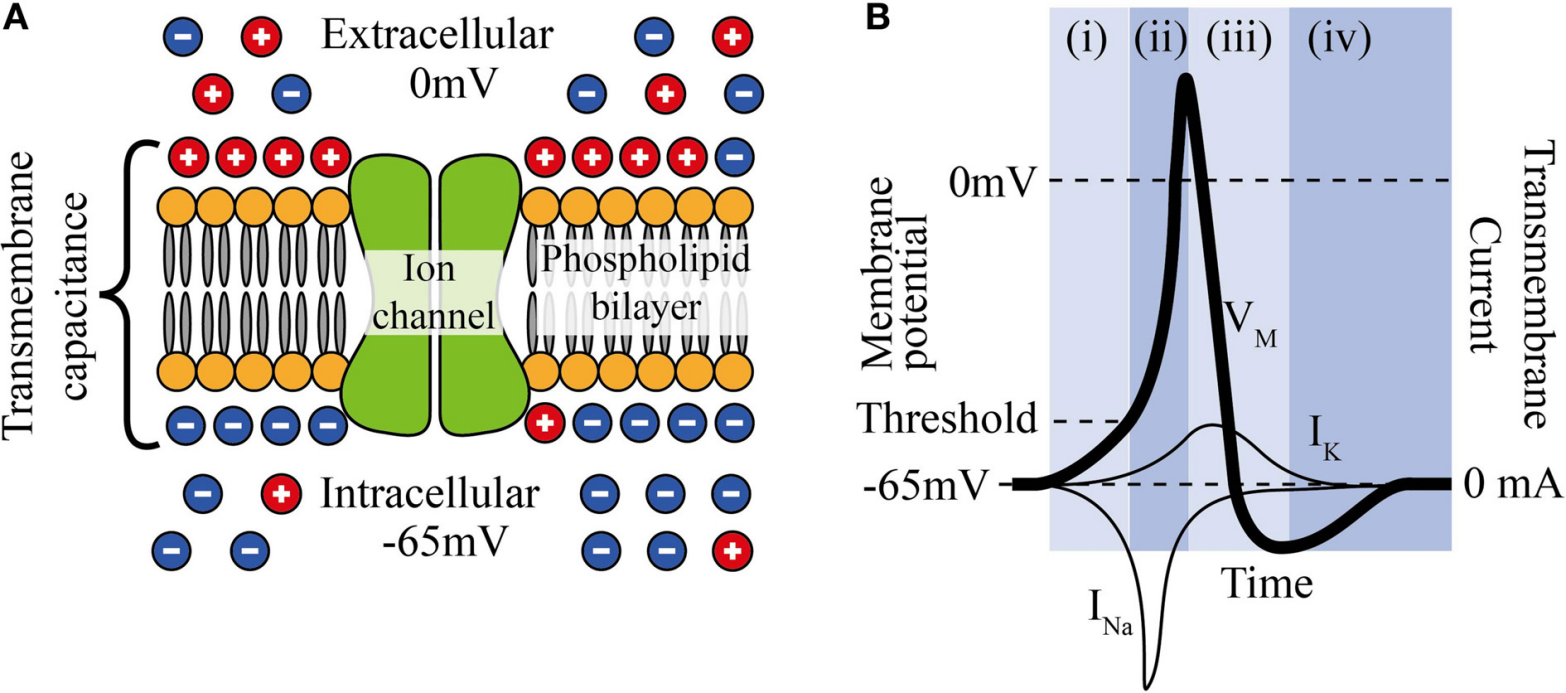
**(A)** The phospholipid cell membrane, ionic charges and an ion channel. **(B)** A typical action potential (i) stimulation causing depolarization to above threshold, (ii) Na^+^ channels open and Na^+^ enters cell, (iii) K^+^ channels are open and K^+^ leaves cell, (iv) ion pumps restore resting potential.

Neuronal cell membranes consist mainly of a phospholipid bilayer across which selective ion pumps work to create a separation of charge, ultimately resulting in a resting cell membrane polarization where the intracellular potential is between 60 and 80 mV below the extracellular fluid and where the bilayer acts as the dielectric of a capacitor (Figure 1A).

Neural stimulation works by causing a depolarization of part of the cell membrane. If this depolarization reduces the transmembrane potential to a critical level (threshold), voltage gated sodium ion channels open and a positive feedback loop is created that amplifies a small depolarization (~15 mV) into a full reverse polarization of the membrane (Figure 1B) and an action potential is created. This in turn depolarizes the surrounding membrane and ultimately causes the action potential to propagate along the neuron. The states and dynamics of voltage gated sodium and potassium channels play major roles in neuromodulation. Examples of two types of transmembrane currents: depolarizing (by convention negative current, typically sodium ions, *I_Na_*, or calcium ions) and hyperpolarizing (positive current, typically potassium ions, *I_K_*) are shown in Figure 1B.

**Figure 2:**
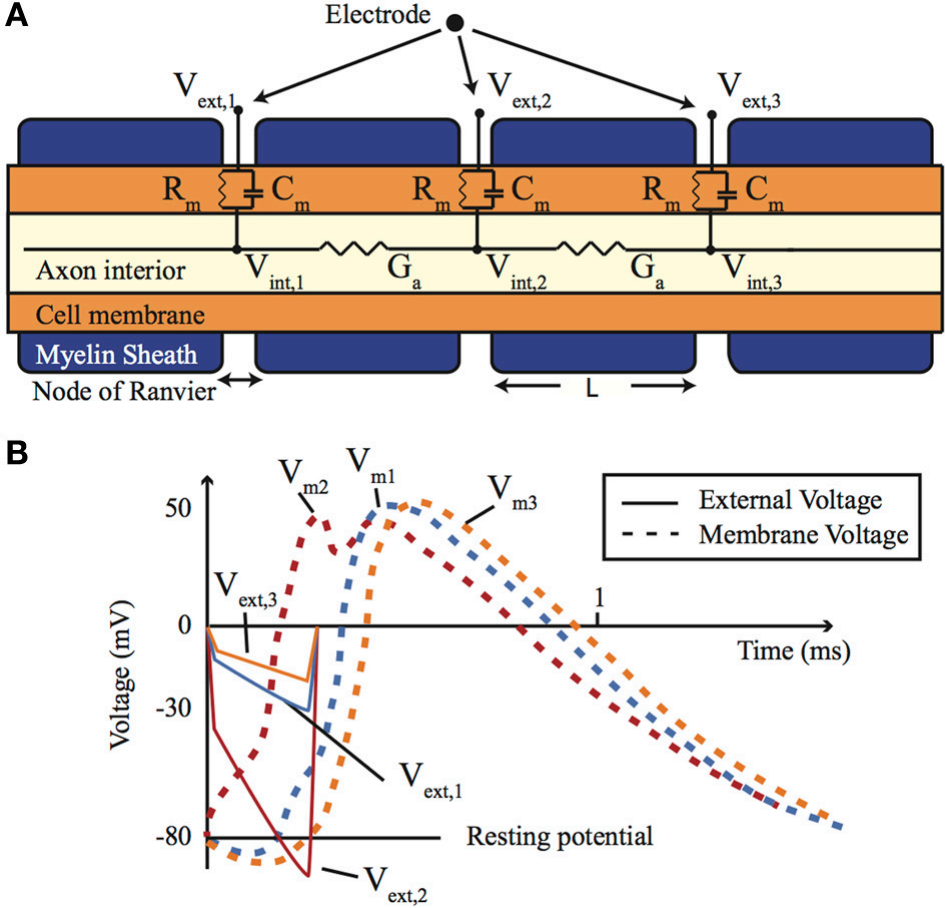
**(A)** A cross-section of a myelinated nerve axon stimulated using an external stimulation electrode and an equivalent circuit model. **(B)** An AP generation in the nerve: simulation on the Neuron platform for AP generation with axon diameter of 15 μm, *L* = 1.5 mm, *T* = 23°C, stimulus = 300 μs, stimulus current of 147 μA. Lines show the extracellular (*V_e_*) and membrane voltage (*V_m_* = *V_i_* − *V_e_*, *V_i_* is intracellular voltage) at the nodes 1, 2, and 3 shown in **(A)**. For more details about the electrode-electrolyte model and the nerve model which is in this case *Xenopus laevis* sciatic nerve see Mou et al. (2012).

When the axon is coated in a myelin sheath (as depicted in Figure 2) the exchange of ions is limited to the gaps in the sheath (the Nodes of Ranvier). This restriction causes action potential to appear to leap from node to node, whilst the myelination of the axon significantly reduces the membrane capacitance and greatly increasing the speed of the AP propagation.

Each neural cell represents an spatially extended electrically active object and in order to model it the cell can be considered to be divided into equipotential compartments (as shown in Figure 2). Then the change of the charge (*Q*) inside a compartment is given by:

(1) $$\frac{dQ}{dt}=-I_{ionic}+I_{intracell}+I_{injected}$$

where *I_ionic_* represents the currents through the ion channels and pumps into that compartment, *I_intracell_* is the internal cell current influx from neighboring compartments, and *I_injected_* are externally injected currents. Replacing *Q* = *C_m_U* in Equation (1), it becomes a classical Hodgkin-Huxley type Equation (Hodgkin and Huxley, 1952).

(2) $$\begin{array}{l} C_{m}\frac{dV_{m}}{dt}=-\sum_{k}I_{ion,k}+\sum_{i}G_{a,i}(V_{ext,i}-V_{ext})\\ \;+\;I_{injected}-(V_{m}+V_{rest})\frac{dC_{m}}{dt} \end{array}$$

where *C_m_* and *V_m_* are the membrane capacitance and the reduced membrane voltage of the considered cell compartment (we drop any index to label it in order to simplify equations), and *V_m_* = *U* − *V_rest_* where *V_rest_* is the resting voltage across the membrane. Also *U* = *V_int_* − *V_ext_*, where *V_int_* and *V_ext_* are the internal and external electrical potential of a compartment. *G_a,i_* is the conductance of axoplasm between the compartment *i* and the considered compartment. The ionic current of the type *k* in Equation (2) is given by:

(3) $$I_{ion,k}={\bar{g}}_{k}\xi^{p_{k}}\eta^{q_{k}}(V_{m}-E_{k})$$

where *g*_*k*_ is the maximum conductance of the ion channels relevant for this ionic current, ξ and η are the ion channel activation and inactivation variables respectively, *p_k_* and *q_k_* are the exponents which model these gating mechanisms, and *E_k_* is the reversal potential of the channel. All activation (ξ) and inactivation (η) variables can be described by differential equations of the form:

(4) $$\frac{d\xi }{dt}=\left[\alpha_{k}(V_{m},t)\cdot (1-\xi )-\beta_{k}(V_{m},t)\cdot \xi \right]$$

where α_*k*_(*V_m_*, *t*) and β_*k*_(*V_m_*, *t*) are voltage and time-dependent rate functions. They can also depend on the concentration of certain ion species (such as Ca^2+^, for example for calcium dependent potassium ion channels). In the case of optogenetic ion channels (such as channelrhodopsin, halorhodopsin, etc) they depend on the light flux as well. Furthermore they can depend on the concentration of various neurotransmitters in the case when Equations (3, 4) describe synaptic currents.

Generally speaking both membrane kinetics and the channel conductance (permeability) are affected by temperature (Hodgkin and Huxley, 1952; Huxley, 1959). Hence both Equations (3, 4) may include a thermic coefficient ϕ, introduced as a multiplier of the expression on the right-hand side of these equation. The thermic coefficient depends on the difference between the actual temperature *T* and a referent temperature *T*_0_ and was first introduced by Hodgkin and Huxley (Hodgkin and Huxley, 1952; Huxley, 1959) to describe the acceleration in the membrane kinetics. It usually takes the form:

(5) $$\phi =Q_{10}^{(T-T_{0})/10}$$

where *Q*_10_ is a special constant corresponding to the kinetics and permeability increase when the temperature is increased by 10°C.

Note that the model described by Equation (2) assumes that the extracellular potential that is produced by the neuron's own activity is negligible. Furthermore, it is assumed that the ionic pumps quickly restore the ionic gradients across the membrane so that the reversal potentials in Equation (3) are constant (otherwise the Goldman-Hodgkin-Katz equation should be used for the ionic currents).

## 3. Neuromodulation modalities

Based on Equation (2), a depolarization can be initiated in a number of ways. This section describes 6 classes of neuromodulation (including the principle of operation and current methods of implementation) and contrasts key parameters in Table 1.

**Table 1:** **Comparison of different neuromodulation modalities**.

	**Direct electrical**			**Magnetic**		**Opto-genetic**	**Thermal**		**Acoustic/ mechanical**	**Chemical**	
	**VCS^a^**	**CCS**	**QCS**	**TMS**	**μMS**		**Optically induced**	**Nano-particles**		**Neuro-transmitter**	**Ion conc**.
Typical stimulus power/electrode	~100 μW			~1 kW		100 mW/cm^2^	~1 kW/cm^2^^b^	100 W	~10 mW/cm^2^^b^	~100 μW	~1 μW
	Efficiency:VCS > QCS >CCS										
AP latency	<1 ms			~1 ms	~10 ms ^c^	~1-10 ms	~1 ms	~10 s	~10 ms	~1 s^d^	~1 min
Spatial resolution	Limited by electrode (e.g., size, charge capacity^e^, ^f^)			~1 cm	~100 μm	Sub-Cellular	~10μm	Single Cell	~0.1–1 mm	Depends on chemical diffusions, etc.	
Invasiveness	Either	Either	Yes	No	Yes	Either	Yes	Yes	No	Yes	Yes
Genetic preparation	No	No	No	No	No	Yes	No	Yes	No	No	No
Typ. system size	~1 mm			~10 cm		~1 mm	~1 cm	~10 cm	~10 cm	~1 mm	
Typ. size of electrode/coil/aperture	~100 μm			ø ~1 cm	ø ~1 mm	~100 μm	~100 μm	ø ~1 mm	~1 mm	~10 μm	
Use on human	Yes			Yes	No	No	No		No	No	
(Potential) Health Risk	Electrochemical reaction			Stroke, eddy current	Not clear	Photo-toxicity/ bleaching	Thermal tissue damage	NP toxicity	Standing wave	Neuro-transmitter leakage	Electro-chemical reaction
Cellular specificity	Stimulation waveform profile		Not clear	Not Clear	Orientation	Gene-targeting	Not clear	Protein-targeting	Not clear	Neuro-transmitter level	Not clear
Target nervous system	CNS/ PNS	CNS/ PNS	CNS/ PNS	CNS/ PNS	CNS/ PNS	CNS	CNS/ PNS	CNS	CNS	CNS	CNS/ PNS
Main/potential applications	DBS	RI, CP	ISMS	Psychiatry	Research	RI, DBS, Research	CI	Research	RI, CI	RI, CP	Facilitate ENS
Engineering challenges for miniaturization	Current control	Energy efficiency	Sensitive to parasitic capacitances	Large devices	High power driver	Genetic modification	High power	Toxicity	High power	Complex fabrication	

a VCS, Voltage Controlled Stimulation; CCS, Current Controlled Stimulation; QCS, Charge Controlled Stimulation; TMS, Transcranial Magnetic Stimulation; AP, Action Potential; CNS/PNS; Central/Peripheral Nervous System; DBS, Deep Brain Stim.; RI, Retinal Implant; CI, Cochlear Implant; CP, Cortical Prostheses; ISMS, Intraspinal Microstimulation; NP, Nano particles.

b Spatial-peak temporal-average intensity.

c <*1* ms if coil is perpendicular to the plane of the neuron; ~10 ms if parallel.

d Can be improved to ms using electro-osmosis.

e Minimum required charge quantity for stimulation determines the minimum surface area of the electrode as electrodes have a a maximum recommended charge capacity per unit area. Cogan (2008).

f *Transcranial Direct Current Stimulation (tDCS) is a non-invasive method for stimulation and has a poor resolution*.

### 3.1. Direct electrical

Direct electrical stimulation uses electrodes to apply a potential gradient across a neuron (e.g., differing extracellular potentials at nodes *V*_*ext*,1_, *V*_*ext*,2_, and *V*_*ext*,3_ in Figure 2 (return electrode is placed in a distant area), which are initially closely followed by the intracellular potentials) causing intracellular ionic current flow (*I_intracell_*) and localized depolarization and hyperpolarization of the cell membrane (*V*_*m*1_, *V*_*m*2_, and *V*_*m*3_ in Figure 2B) that results in neural stimulation. Applying a different potential gradient can be used to create a region of cell membrane hyperpolarization sufficient to block action potential propagation, thereby achieving neural inhibition. More complex stimulation, inhibition and selectivity mechanisms are also possible by applying waveforms that exploit the differing time constants of the ion channels—for example anode break stimulation occurs when a long hyperpolarizing (inhibitory) pulse is suddenly ceased and results from differences in the rate that the sodium activation and inactivation gates change state (Zhou and Greenbaum, 2010).

Key considerations for direct electrical stimulation are safety, energy efficiency, area, spatial resolution and programmability. Safety is largely determined by two key characteristics: (1) the electrode material biocompatibility, and (2) minimizing the net creation of harmful electrochemical products. This latter criteria is typically achieved by limiting the rate and total amount of charge delivered through the electrodes, restricting the maximum potential difference across the electrodes and minimizing the residual charge left on the electrodes following stimulation. Commercial products use large DC blocking capacitors to limit this residual charge, improving safety at the expense of size and weight (which limits the number of channels that can be implemented). As a result, designs free of blocking capacitors are an active area of research (Sit and Sarpeshkar, 2007; Liu et al., 2008). Electrode size (typical diameters are in the order of 0.1 mm) is important in determining spatial resolution, however, minimizing the electrodes is not only limited by fabrication capability but also the required charge to be delivered and the maximum safe charge that an electrode can deliver per unit surface area (a function of the electrode surface material). In practice spatial resolution is also limited by how close the electrode is to the target neural tissue. As this distance increases the stimulation strength must be increased in order to remain effective—causing a larger activation volume and stimulation of other non-target neurons. The time between stimulation onset and action potential generation (AP latency) depends on the activation time constant of the voltage gated ion channels. It varies with neuron and ionic types and is typically in the order of milliseconds. There is a trend in research toward increasing stimulator programmability—providing control over all the parameters related to the stimulus waveform—which potentially allows changes to stimulation efficacy without changing stimulator or repositioning the electrodes (Macherey et al., 2006; Wongsarnpigoon and Grill, 2010).

**Figure 3:**
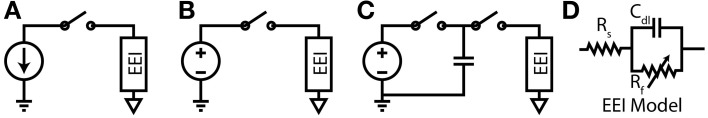
**(A)** Current-Controlled Stimulation **(B)** Voltage-Controlled Stimulation **(C)** Charge-Controlled Stimulation **(D)** Electrode-Electrolyte Interface model [Rs is solution spreading resistance, *C*_*dl*_ is double layer capacitance, *R_t_* is charge transfer resistance (Luan and Constandinou, 2012)]

The required potential gradients for stimulation can be generated using Voltage, Current or Charge controlled stimulators (see Figure 3).

#### 3.1.1. Voltage controlled

Directly applying a voltage between two electrodes is the simplest and lowest power method of direct neural stimulation, however, the lack of control of charge delivered to the electrodes leads to electrode degradation and toxic redox products. Clinically these stimulators are used in Deep Brain Stimulation (Hardesty and Sackeim, 2007) or muscular stimulation [e.g., pacemakers (Wong et al., 2004)] where the low power consumption and previously demonstrated therapeutic efficacy are the dominant considerations.

#### 3.1.2. Current controlled

In current controlled stimulation the current flowing between two electrodes is controlled by applying a time varying potential difference across the electrodes. Controlling current enables charge balanced waveforms to be used (e.g., by careful calibration of current sources, use of an H-bridge; or series DC blocking capacitors) and the charge delivered to the electrodes can be constrained within their charge capacity. However, controlling the current leads to significant power waste with implications for tissue damage and battery lifetime. Clinically this approach is used in cochlear implants and is the most popular method in research publications (Thurbon et al., 1998; Srinivasan et al., 2010).

Transcranial Direct Current Stimulation (tDCS) has recently been used to study the physiology of the CNS in human (Nitsche and Paulus, 2000). It is a safe, non-invasive method, but with low spatial resolution (in common with other non-invasive methods). Yet, Horvath et al. (2014) points out that more research is needed before drawing a useful conclusion on the efficacy and reliability of tDCS.

#### 3.1.3. Charge controlled

Charge controlled stimulation combines voltage stimulation with a capacitor to limit or control the charge delivered to the electrodes (Ghovanloo, 2006; Bawa, 2008; Rosellini et al., 2011; Luan and Constandinou, 2012). In terms of biocompatibility and power consumption, this approach offers a mid-ground between current and voltage controlled stimulation, however, it is not yet been reported to be in clinical use.

### 3.2. Magnetic

The mechanism for magnetic stimulation is similar to that of direct electrical stimulation, except that the potential gradients are induced in the tissue by a rapidly changing strong magnetic field (>1 T)—typically generated by discharging large capacitors through an electromagnet or via kilowatt amplifier.

It is usually implemented transcutaneously [e.g., Transcranial Magnetic Stimulation (TMS)] and hence non-invasively. The removal of the electrochemical issues associated with the electrode, potentially offers a major advantage over electrical stimulation, however, these devices currently offer poor spatial selectivity and high peak power consumption. The improvement of stimulus focality mainly relies on the redesign of the stimulation coil. Recently, Bonmassar et al. (2012) reported on the design of a micro-TMS (μTMS) system which reduces the spatial resolution by an order of magnitude. However, the large power consumption is an obstacle for building a fully implantable stimulator.

Hand-held TMS devices have been used for neuroscience purposes and clinically for stroke and depression treatment. However, cautions must be paid when using TMS with any metallic implant like pacemaker, where the metal sheet could be heated by the eddy current generated during TMS. Reviews on TMS can be found in Rossini et al. (2010) and in combination with tDCS as two main methods for exploring the dynamics of the human CNS in Dayan et al. (2013).

### 3.3. Optogenetic

**Figure 4:**
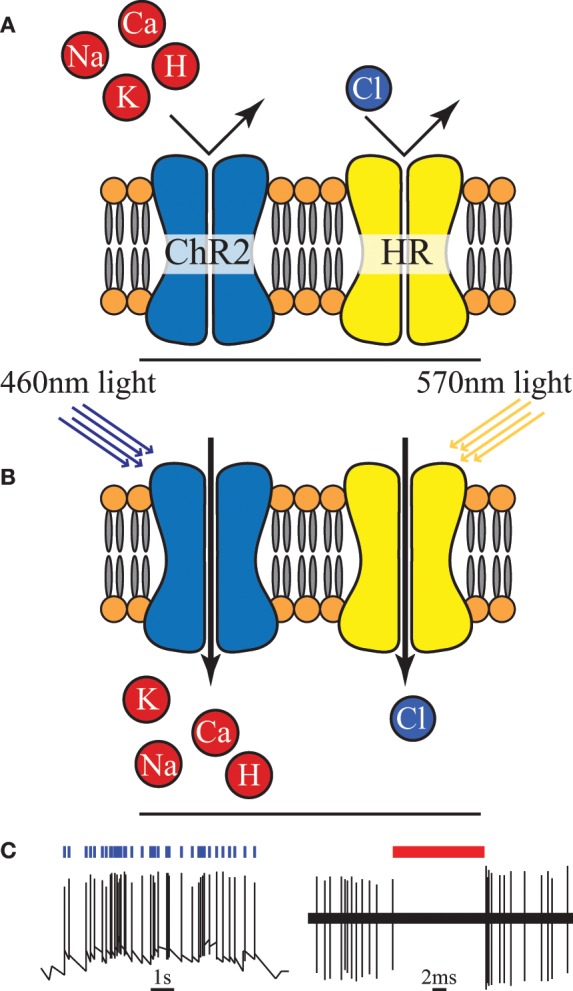
**(A)** An unilluminated channelrhodopsin-2 (ChR2) ion channel and halorhodopsin (HR) ion pump are closed and inactive. **(B)** Once exposed to light of specific wavelength, ChR2 allows certain positive ions into the cell, and HR begins to pump chloride ions in. **(C)** Activation of ChR2 initiates individual action potentials, in contrast HR activation suppresses action potentials, redrawn from Boyden et al. (2005) and Chow et al. (2010).

Optogenetics is a relatively new neuromodulation technology in which light-sensitive proteins (“opsins”) are genetically inserted into cell membranes or cells (Nagel et al., 2003; Boyden et al., 2005). These proteins act as light-activated ion pumps, channels or enzymes to achieve fast and precise optical manipulation of electrical and biochemical processes in cells as well as modulation of signaling cascades (Yizhar et al., 2011). Optogenetics has numerous applications which are only beginning to be explored—hence its choice as “Method of the Year 2010” by Nature Methods (Deisseroth, 2011). The first and most famous of these opsins were channelrhodopsin for neural stimulation (Nagel et al., 2003) and halorhodopsin for neural inhibition (Chow et al., 2010) (see Figure 4). Since then a wide range of opsins with varying temporal, chemical and spectral properties have been discovered. This has been combined with parallel development of techniques for genetic manipulation and improvements in our understanding of gene expression; ultimately resulting in a tool with peerless precision for neural manipulation.

However, creating a chronic, portable implant capable of exploiting even a fraction of the potential of this technique remains an open challenge (Williams and Denison, 2013). *In-vivo* experimental setups to date have typically involved either benchtop lasers (coupled with fiber-optics) or transcutaneous illumination. However, McCall et al. (2013) have taken a major step in demonstrating a fully wireless system of up to 25 channels albeit with only a fraction of the feasible targeting precision. The need to genetically modify the neurons is a major drawback in applying this technology in humans, both from a safety and ethics perspective (especially in the brain and CNS in general) and in clinical applications in the PNS (where it is unclear how quickly new ion channels would be expressed along the long axons that exist in the periphery). On the more technical side a potential source of noise and uncertainty is that cells are typically loaded with a vector and then variations of the gene expression can lead to different responses for the same stimulus. However, the high precision and ability to selectively express the opsins in target neuron types makes this a highly attractive tool for neuroscience [especially for decoding brain circuitry (Yizhar et al., 2011)] and for retinal prosthesis development (Busskamp and Roska, 2011).

### 3.4. Thermal

Thermal modulation of neural activity is believed to result from a combination of the temperature induced changes in the transmembrane capacitance (the last term in the RHS of Equation 2) and non-uniform changes in the conductance dynamics of the various ionic channels (Equations 3, 4) (Duke et al., 2013; Peterson and Tyler, 2014).

During rapid localized heating, the capacitive effect dominates and the reduced transmembrane capacitance leads to ionic current flow, depolarization and action potential initiation (Shapiro et al., 2012; Duke et al., 2013). In this case the last term in Equation (2) creates a stimulus proportional to the speed of temperature change (*dT*/*dt*, because *dC_m_*/*dt* can be expressed as: *dC_m_*/*dt* = (*dC_m_*/*dT*) · (*dT*/*dt*). The plasma membrane electrical capacitance is reversibly affected by the temperature (*dC_m_*/*dT* term) because the total capacitance of a lipid membrane in electrolyte solution includes in-series capacitance of ionic double layers at the interface between the (polar) membrane surface and electrolyte (Shapiro et al., 2012). Conversely during slow heating the changes to ionic channels dominate and in particular changes to Na^+^ and K^+^ activation/deactivation dynamics prevent action potential initiation and propagation (Mou et al., 2012; Duke et al., 2013). Thermal damage to tissue is a major issue with this method of neuromodulation, especially for suppression due to the higher temperatures and slower, more diffuse heating necessary (Wells et al., 2007).

There are a number of ways of inducing this thermal change, here we cover optical and nanoparticle stimulation. Bench-top equipment has previously been required, however, an emerging method using a CMOS lab-on-chip micro-heater array is recently reported in Reverter et al. (2014).

#### 3.4.1. Optically induced thermal modulation

Rapid heating can be achieved through the targeting of near infra-red laser light on a neuron or nerve (Wells et al., 2005; Duke et al., 2013). This enables a typical AP latency of the order of ms and a high spatial resolution of the order of 10 μm. But effective stimulation and damage thresholds are within an order of magnitude for some neurons which is potentially a cause for concern (Richter et al., 2011). Laser induced heating has also been demonstrated for neural inhibition as part of a hybrid electrical stimulation/thermal inhibition device (Mou et al., 2012; Duke et al., 2013).

#### 3.4.2. Microwave/RF heating of nanoparticles

Magnetic nanoparticles can absorb RF radiation and therefore heat surrounding tissue. Combining these particles with proteins known to bind to specific protein targets on neural cell membranes has been shown to enable focused heating of target cells. Although this approach could potentially give very good spatial resolution, the temporal resolution and power consumption of this technique remain poor and the safety of the nanoparticles is unclear (Huang et al., 2010).

### 3.5. Acoustic/mechanical

Acoustic modulation is an emerging method. Experiments have indicated that on-off modulated ultrasound waves can elicit action potentials from retinal and brain cells (Tufail et al., 2010; Naor et al., 2012; Menz et al., 2013). However, very little is understood about how mechanical deformations (typically in the cell membrane) affect ion channels, membrane capacitances and neurons—although proposed mechanisms have included cavitation and thermal effects. Despite a lack of clarity on the actual mechanism, potential applications for non-invasive Deep Brain Stimulation and retinal prostheses are being investigated.

The main benefit of this method is that it is non-invasive and is likely to offer better spatial resolution than other non-invasive techniques—particularly useful for applications like retinal prostheses (Menz et al., 2013). The drawbacks of the acoustic method is that the standing waves could also stimulate unwanted neural tissue.

### 3.6. Chemical

Microfluidics can be used to control the chemical environment around a neuron, potentially changing its transmembrane or post-synaptic potential and inducing or suppressing action potential generation. Achieving high temporal or spatial resolution with such systems may be challenging—depending on concentrations required to elicit effects and the dynamics of the chemical diffusion, breakdown or uptake in the tissue. However, it is an approach that may be suited to applications requiring a longer term effect with a wider area of effect, e.g., Deep Brain Stimulation. Major challenges with this approach include safe chemical storage, modulation efficacy and economic manufacture.

#### 3.6.1. Neurotransmitter modulation

Neurotransmitter molecules released near the synapses between two neurons, bind to the receptors in the post-synaptic cleft—altering the post-synaptic potential or initiating a chemical change that affects neuronal signal transmission. The selective sensitivity of neurons to specific neurotransmitters means that specific types of neurons can potentially be targeted even in a heterogeneous environment (like the retina) (Theogarajan, 2012). However, this approach has limited applicability in the PNS where synapses are absent or only present at the terminus of the axon which is often too diffuse a target for implantation.

#### 3.6.2. Ionic concentration

Sequestration or release of anions or cations near a neuron disrupts the diffusion and potential gradients across the cell membrane and acts to modulate neural transmission. This can be used for standalone inhibition or to reduce required stimulation thresholds in tandem with another stimulation technique (Song et al., 2011). The use of ions rather than neurotransmitters widens the applicability of this technique, enabling it to be used in the PNS but conversely reduces its specificity.

## 4. Discussion

**Figure 5:**
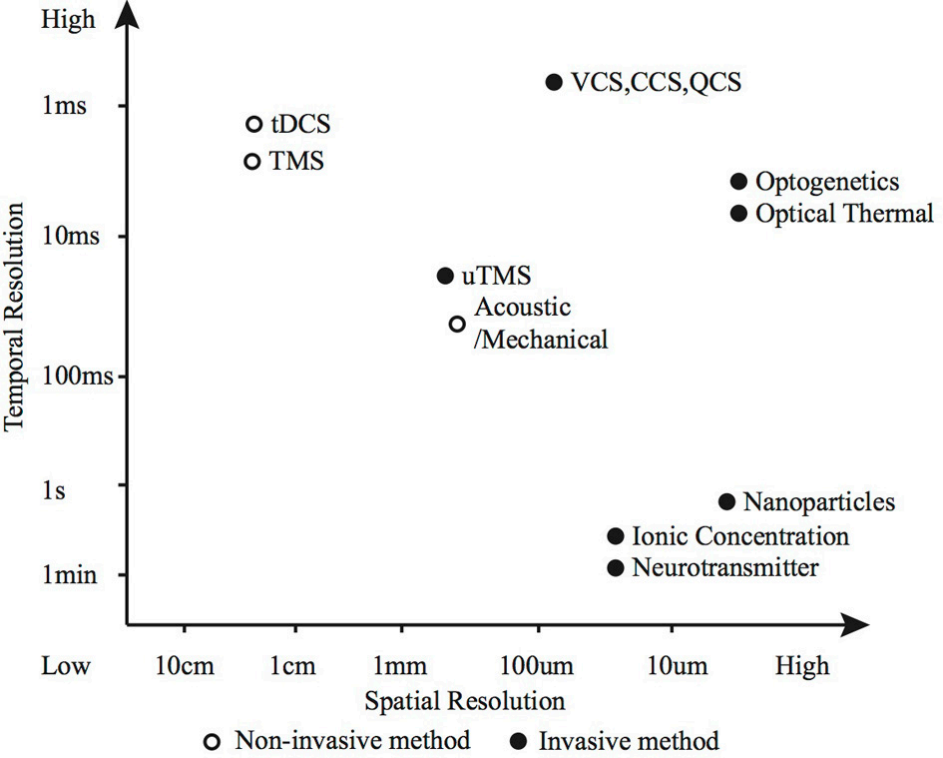
**Spatio-temporal resolution of various neuromodulation methods**. The open circle represents non-invasive methods and the black dots invasive methods.

Choosing a neuromodulation method for clinical application is guided by 3 main considerations:

1. The safety of a modulation method (including tissue damage, side effects and ethical considerations) are of primary importance. All the techniques discussed here have the potential to cause harm if misapplied, however, optogenetics and nanoparticle based thermal modulation stand out as facing particularly tough challenges in making the leap to human clinical use, due to genetic modification and nanoparticle concerns.
2. The efficacy of each modulation method for a particular application is dependent on: the required modulatory effect; the spatial, temporal and cellular resolution; and the biological characteristics of the modulation site. Precise control of action potential timing and frequency (typically desired for sensory and motor prostheses) is suited to electrical or optogenetic methods (Figure 5 shows how various methods compare in terms of spatio-temporal resolution); whereas larger scale, unfocused neural activity suppression or promotion (such as brain stimulation for tremor, depression or epilepsy) is better suited to electrical, magnetic, chemical or potentially thermal nanoparticle methods.
3. The suitability to a specific application use case (e.g., the frequency, duration and range of situations in which modulation will be applied) is largely dependent on requirements for acute or chronic use. Short term use in a clinical setting leads to a strong preference for a non-invasive solution such as transcranial electrical or magnetic modulation or potentially an acoustic/mechanical solution. On the other hand, long term, frequent modulation means portability is a priority and invasive solutions become more acceptable—currently favoring electrical modulation.

There is a good reason why electrical neural stimulation is the most widely used today. It is the most mature of the stimulation technologies with low AP latency and capable of a wide range of spatial resolutions that satisfy most applications. Microelectronic fabrication can already produce stimulators that are small, implantable, low power and safe, and research is underway to improve the stimulation efficiency, efficacy and to integrate more channels onto a single chip. However, ENS is not without its limitations, and these represent an opportunity for competing methods to prosper.

This review has identified a wide diversity of available neuromodulation techniques which, although less mature, each demonstrate unique strengths and have the potential to supplant ENS in specific applications. For example, in deep brain stimulation, spread of electrical stimulus has been linked to side-effects such as depression. A lot of research has been dedicated to design a new type of electrode which can shift and confine the stimulus within a certain area, however, pursuing other neuromodulation methods that provide greater spatial resolution or cellular specificity may be an alternative solution—e.g., chemical modulation using neurotransmitter release (offering a degree of cellular specificity), or optogenetics (with high spatial resolution and cellular specificity) may one day be viable options. The shift from ENS is already well underway in non-clinical applications, where optogenetics is a frequently favored method. Nano-particle based thermal stimulation and neurotransmitter chemical stimulation are also alternatives that offer cellular specificity and may find successful niches in this area.

Looking to the future, we see a trend for multimodal neuromodulation—combining the advantages and mitigating the disadvantages of different modalities. Examples include combining current stimulation with voltage control to reduce power (Williams and Constandinou, 2013), voltage stimulation with current control (Luan and Constandinou, 2012), hybrid electro-optical stimulation (Duke et al., 2012; Mou et al., 2012), or integrating current controlled stimulation with microfluidics to reduce stimulation thresholds (Song et al., 2011). Electrical neural stimulation remains the gold standard in clinical use (especially for chronic use or in the PNS), however, for applications demanding increased efficacy, increased modulation complexity and reduced side-effects the days of using just electrical stimulation on its own may be numbered.

### Conflict of interest statement

The authors declare that the research was conducted in the absence of any commercial or financial relationships that could be construed as a potential conflict of interest.
